# A New Chalcone Derivative C49 Reverses Doxorubicin Resistance in MCF-7/DOX Cells by Inhibiting P-Glycoprotein Expression

**DOI:** 10.3389/fphar.2021.653306

**Published:** 2021-04-13

**Authors:** Ting Wang, Jingjing Dong, Xu Yuan, Haotian Wen, Linguangjin Wu, Jianwen Liu, Hua Sui, Wanli Deng

**Affiliations:** ^1^ Department of Medical Oncology, Putuo Hospital, Shanghai University of Traditional Chinese Medicine, Shanghai, China; ^2^ Shanghai Bailijia Health Pharmaceutical Technology, Shanghai, China; ^3^ Shuguang Hospital, Shanghai University of Traditional Chinese Medicine, Shanghai, China; ^4^ State Key Laboratory of Bioreactor Engineering and Shanghai Key Laboratory of New Drug Design, School of Pharmacy, East China University of Science and Technology, Shanghai, China; ^5^ Medical Experiment Center, Jiading Branch of Shanghai General Hospital, Shanghai Jiao Tong University School of Medicine, Shanghai, China

**Keywords:** breast cancer, DOX, C49, multidrug resistance, P-gp, PI3K/akt signaling pathway

## Abstract

**Objective:** C49 is a chalcone derivative. The aim of the current study is to illuminate the efficacy of C49 in reversing multidrug resistance (MDR) in MCF-7/DOX cells and its underlying molecular mechanism.

**Methods:** The cytotoxic effects of C49 on MCF-7/DOX cells were evaluated by MTT assay using different concentration (0–250 μmol/L) of C49. Cell proliferation was evaluated by colony formation assay. Cell death was examined by morphological analysis using Hoechst 33,258 staining. Flow cytometry and immunofluorescence were utilized to evaluate the intracellular accumulation of doxorubicin (DOX) and cell apoptosis. The differentially expressed genns between MCF-7 and MCF-7/DOX cells were analyzed by GEO database. The expression of PI3K/Akt pathway proteins were assessed by Western blot The activities of C49 combined with DOX was evaluated via xenograft tumor model in female BALB/c nude mice.

**Results:** C49 inhibited the growth of MCF-7 cells (IC_50_ = 59.82 ± 2.10 μmol/L) and MCF-7/DOX cells (IC_50_ = 65.69 ± 8.11 μmol/L) with dosage-dependent and enhanced the cellular accumulation of DOX in MCF-7/DOX cells. The combination of C49 and DOX inhibited cell proliferation and promoted cell apoptosis. MCF-7/DOX cells regained drug sensibility with the combination treatment through inhibiting the expression of P-gp, p-PI3K and p-Akt proteins. Meanwhile, C49 significantly increased the anticancer efficacy of DOX *in vivo*.

**Conclusion:** C49 combined with DOX restored DOX sensitivity in MCF-7/DOX cells through inhibiting P-gp protein.

## Introduction

Breast cancer has the highest mortality of female cancers and is the second cause of death in females (Siegel et al., 2020). Treatment for breast cancer includes surgery, chemotherapy, and radiotherapy, among them, chemotherapy plays a critical role (Harbeck et al., 2017; Waks and Winer, 2019). The first-line chemotherapeutic drug for breast cancer is doxorubicin (DOX), which is an anthracycline (Li et al., 2019b; Pondé et al., 2019; Tun et al., 2019; Zeinoddini et al., 2019). DOX represses DNA replication, interrupts cell cycle, and facilitates generation of intracellular reactive oxygen species (Meredith et al., 2016; Cui et al., 2018; Khaki-Khatibi et al., 2019) to induce tumor cell death (Marinello et al., 2018; El-Hamid et al., 2019). However, breast cancer cells generate DOX resistance and cause serious cardiotoxicity with increasing length of chemotherapy. These two factors are major causes for treatment failure and metastasis of breast cancer (Cappetta et al., 2017; Li et al., 2017; Ponnusamy et al., 2018; Wenningmann et al., 2019; Zheng et al., 2019; Al-Malky et al., 2020).

Numerous studies have found that P-glycoprotein (P-gp) expression is elevated in breast cancer patients who are insensitive to chemotherapy (Badowska-Kozakiewicz et al., 2016; Dyson et al., 2018; Mehrotra et al., 2018). This finding indicates that P-gp on cell membrane may participate in the development of drug resistance in breast cancer (Pokharel et al., 2016; Ge et al., 2017; Xiong et al., 2018). P-gp is a transmembrane protein encoded by ABCB1 gene, which is an ATP-dependent drug transport protein and is closely associated with multidrug resistance (MDR) of tumors. The function of P-gp protein is to excrete endogenous and exogenous substances and reduce the content of intracellular chemotherapeutics, as a result, chemotherapeutics fail to effectively kill tumor cells (Waghray et al., 2018; Guo et al., 2019). This defense mechanism is important for tumor cells to evade chemotherapeutic attack (Kartal-Yandim et al., 2016). A number of studies show that MDR can be reversed by inhibiting P-gp transport activity or competitively binding to P-gp protein binding site with chemotherapeutics (Li et al., 2016; Komoto et al., 2018; Chang et al., 2019).

Although several drugs can reverse drug resistance by repressing P-gp protein, their side effects have restricted the clinical application. For instance, verapamil may give rise to cardiotoxicity, and cyclosporin A may cause hepatotoxicity, nephrotoxicity, myelogenous toxicities and neurotoxicity (Joshi et al., 2017; Ding et al., 2018; Dong et al., 2019). Therefore, there is no approved MDR-reversing agent applied in clinical chemotherapy of cancers available at present. Thus, developing drugs with efficient reversal activity and few toxicities is urgently needed.

Chalcone derivatives extensively exist in plants, such as *glycyrrhiza* and *lupulus* (Watanabe et al., 2016; Gomes et al., 2017; Seliger et al., 2018; Wu et al., 2020). They have multiple biological activities, such as antioxidation and antivirus (Mateeva et al., 2017; Liang et al., 2018; Gupta et al., 2019; Lin et al., 2019; Maria Pia et al., 2019; Xu et al., 2019). Some researchers have designed a quinone chalcone compound, which can inhibit DNA topoisomerase I and has anti-inflammatory activity. The compound has a strong anti-tumor effect, and its inhibition rate on breast cancer cells is as high as 50% at the dose of 10 μg/ml. Natural chalcones have few side effects and high anti-tumor activity. Therefore, finding new natural chalcone derivatives has attracted a lot of interests. Studies have shown that chalcone derivatives have antitumor activity, such as facilitating apoptosis and autophagy of hepatocellular carcinoma cells and inhibiting proliferation of human bladder cancer cells (Wang et al., 2017; Hong et al., 2019; Pinto et al., 2019; Yang et al., 2019; Zhu et al., 2019). Previous studies have reported that some chalcone derivatives can repress the expression of P-gp protein, increase DOX accumulation in cells, and reverse MDR (Yin et al., 2019). In this study, we investigate a new chalcone, namely C49, for its ability to enhance the chemosensitivity of MCF-7/DOX cells to DOX and the possible mechanism of action. This study will provide a novel therapeutic option for breast cancer.

## Materials and Methods

### Cell Culture

MCF-7/DOX and MCF-7 cells were obtained from the Cell Bank of Chinese Academy of Science (Shanghai, China). The cells were cultured in RPMI1640 medium containing 10% fetal bovine serum (FBS) at 37°C, in a humidified atmosphere with 5% CO_2_. The resistance of MCF-7/DOX cells was maintained by using a medium containing DOX at a concentration of 50 ng/ml.

### Reagents and Chemicals

C49 (>99% purity) was synthesized in the School of Pharmacy of East China University of Science and Technology. DOX, 3-(4,5-Dimethylthiazol-2-yl)-2,5-diphenyltetrazolium bromide (MTT), and Hoechst 33,258 were purchased from Sigma Chemical Co. The primary antibodies, P-gp, Caspase-3, Caspase-9, Caspase-10, Bcl-2, Bcl-xL, p53, phospho-p53, PI3K, phospho-PI3K, Akt, phospho-Akt, β-actin, and Ki-67, were brought from Proteintech.

### Synthesis of C49

The method was performed as described previously (Tseng et al., 2013). Ccarboxylic acid (1.47 g, 5.0 mmol) was heated at 280°C for 4 h (TLC monitoring) and add hexane (50 ml), then the resulting precipitate was collected and purified by flash chromatography on silica gel (hexane/CH2Cl2 1/1). During extracted with CH2Cl2 and ethyl acetate (50 ml × 3), the crude product was purified and crystallized with EtOH to give quinolinyl chalcones.

### MTT Cell Viability Assay

The cell viability assays were performed by MTT method (Wang et al., 2016; Śliwka et al., 2016; Schröder et al., 2019). MCF-7 and MCF-7/DOX cells were seeded in 96-well plates (1 × 10^5^ cells/well) until they attached to the plate. Then, cells were treated with C49, DOX, or their combinations at different concentrations. After 24, 48 and 72 h, MTT assay was performed and the IC_50_ of drug was calculated.

### Evaluating the Effect of Drug Combination

The drug combination index (CI) was calculated by Calcusyn (Biosoft, Cambridge, United Kingdom). CI reflects the synergistic effect of the drug combination (Liu et al., 2016; Bahri et al., 2019). The CI value <1, =1, and >1 represents synergy, addiction and antagonism, respectively.

### Colony-forming Assay

MCF-7/DOX cells were seeded in 24-well plates (100 cells per well). Cells were respectively treated with C49 (25 μmol/L), DOX (8 μmol/L) and their combination. After 7 days, they were washed, fixed, stained with 0.1% crystal violet at room temperature for 20 min, and photographed. ImageJ was utilized for quantitative analysis.

### Hoechst 33,258 Staining for Cell Morphology

MCF-7/DOX cells were inoculated into 12-well plates (approximately 2 × 10^5^ cells/well) for 12 h and were respectively cultured with C49 (25 μmol/L), DOX (8 μmol/L) and their combination for 24 h. Then, they were fixed in paraformaldehyde (4%) for 15 min, stained with Hoechst 33,258 for 15 min with phosphate buffer saline (PBS) at room temperature, washed and photographed by a fluorescence microscope (Nikon, Tokyo, Japan).

### Western Blot Assay

WB assay was performed as described previously (Wang et al., 2019). MCF-7 and MCF-7/DOX cells were treated with C49 (25 μmol/L), DOX (8 μmol/L) and their combination for 24 and 48 h. The cells were washed twice with pre-cold PBS and were lyzed using RIPA lysate buffer with phosphatase inhibitor for 30 min. Then, the crude cell lysates were centrifuged at 12,000 rpm for 10 min at 4°C. Equal amounts of protein were separated with SDS-PAGE and transferred onto PVDF membranes. The resultants were blocked with BSA (50 mg/ml) and incubated overnight at 4°C with the primary antibodies of P-gp, Caspase-3, Caspase-9, Caspase-10, Bcl-2, Bcl-xL, p53, phospho-p53, PI3K, phospho-PI3K, Akt, phospho-Akt and β-actin. The PVDF was washed three times with TBST, incubated with horseradish peroxidase-conjugated secondary antibodies (1:2000) for 2 h at the room temperature, and washed three times with TBST. Protein bands were obtained from WB detection system and quantitated using the ImageJ software.

### Gene Expression Profiles

GEO Series Accession Number GSE24460 (https://www.ncbi.nlm.nih.gov/geo/) contains the data of gene expression, which can be obtained from the database of the National Center for Biotechnology Information. It contains four samples including parental MCF-7 cell line vs. DOX-resistant MCF-7 cell sublines. Biological replicates include two parental controls and two drug resistance, which are independently grown and harvested.

### Screening Differentially Expressed Gene

Limma package, Hochberg False Discovery Rate and Benjamini were used to analyze the gene expression profiles and filter out the differentially expressed genes in MCF-7 and MCF-7/DOX cells using GSE24460. The differentially expressed genes was showed in heatmap, volcano and KEGG plot. The fold change threshold was >2, and the *p* value was <0.05.

### Flow Cytometry

Flow cytometry (FCM) was used to detect cell apoptosis and intracellular DOX accumulation (Sui et al., 2017). The apoptosis was detected following the manual of Annexin V-FITC apoptosis detection kit (Invitrogen). MCF-7/DOX cells were seeded in 6-well plates (1 × 10^4^ cells/well). The cells were treated with C49 (25 μmol/L), DOX (8 μmol/L) and their combination for 48 h, collected and washed with PBS twice. Cells were re-suspended in PBS (250 μL) and then analyzed by FACS using flow cytometer (Becton Dickinson) to detect DOX intracellular accumulation.

### Drug Efflux Fluorescence Microscopy Assay

A FP-6200 spectrofluorometer (Jasco Corp., Tokyo, Japan) was used for measuring the emission spectrum of DOX in the presence and absence of DNA. Studies were carried out as described (Xiong et al., 2005). Briefly, MCF-7/DOX cells were plated in 24-well plates (1 × 10^4^ cells/well). When cells were attached, C49 (25 μmol/L) was used with or without DOX (8 μmol/L) for 48 h. Then, cells were exposed to DOX for 2 h, washed and fixed with 4% paraformaldehyde fixation solution for 15 min. DAPI staining was used to stain the nucleus. Cells were washed and photographed using a fluorescent microscope (Nikon, Tokyo, Japan).

### Tumor Xenografts

Female BALB/c nude mice (4–6 weeks old) were purchased from Shanghai Jiesijie Experimental Animal Company and maintained in a specific pathogen-free environment. The animal facility was authorized by the Ministry of Science and Technology of the PRC. MCF-7/DOX cells (1 × 10^6^) were suspended in 100 μL of PBS and injected into the right flank of nude mice. When the tumor size grew up to approximately 50 mm^3^, the mice were randomly divided into 6 groups (*n* = 5): 1) vehicle control (0.1 mL PBS), 2) C49 (5 mg/kg), 3) C49 (15 mg/kg), 4) DOX (2 mg/kg), 5) DOX (2 mg/kg) combined with C49 (5 mg/kg), and 6) DOX (2 mg/kg) combined with C49 (15 mg/kg). Then PBS and C49 were used by tail intravenous injection, and DOX was used by intraperitoneal injection every 2 days for 30 days. During the treatments, the tumor volumes were recorded using formula (L × D × D) × 0.5, where “L” represents the length and “D” represents the breadth of the tumors. Experiments were terminated at the 30th day, and the animals were anesthetized and sacrificed. Tumors were fixed in 10% paraformaldehyde fixation solution for further analysis.

### Hematoxylin and Eosin Staining and Immunohistochemistry Staining

Histological analysis was performed on tissue samples isolated from mouse xenografts. Sections of 5 µm were cut from paraffin-embedded tissues and were prepared according to standard protocols for H&E (hematoxylin and eosin) and IHC (immunohistochemistry) staining. Images of sections were visualized using a microscope (Nikon, Tokyo, Japan).

### Statistical Analysis

GraphPad Prism Software was used to analyze the experimental data. Values were denoted by the mean ± standard deviation or standard error of the mean. *p* values of *p < 0.05, **p < 0.01, ***p < 0.001, ^#^ *p* < 0.05, ^##^ *p* < 0.01, ^###^ *p* < 0.001 represent significant difference.

## Results

### Effect of C49 and DOX Treatment on MCF-7 and MCF-7/DOX Cells

**FIGURE 1:**
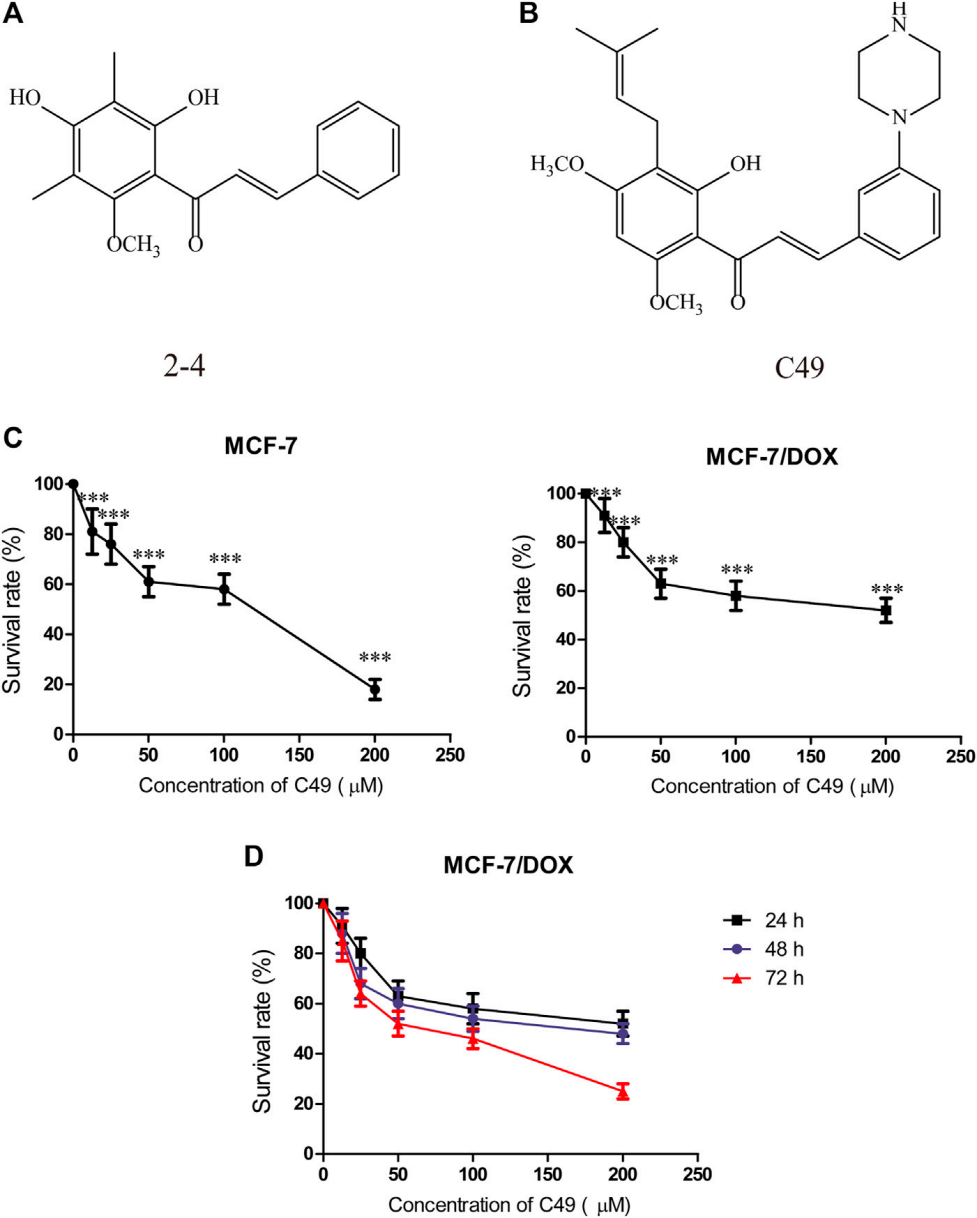
Chemical structure of C49 and the effects of C49 on the viability of MCF-7 and MCF-7/DOX cells. **(A–B)** Chemical structures of C49 and its parent compound. **(C)** MCF-7 cells and MCF-7/DOX cells treated with DOX for 48 h, and the cell viability assessed by MTT assay. **(D)** MCF-7/DOX cells treated with C49 for 24, 48 and 72 h. Viability quantitated by the MTT assay. Each point represents mean ± SD, *n* = 3. *p < 0.05, **p < 0.01, and ***p < 0.001.

C49 is a chalcone derivative, and its structure is shown in Figures 1A,B.

Before we investigated the anticancer efficiency of C49 with different dose of DOX, we first examined the doxorubicin-resistance of MCF-7 and MCF-7/DOX cells by MTT assay at 24 h. As showed in Figure 1C, the results indicated that DOX exerted significantly inhibit effect in MCF-7 cell when the dose of DOX exceed 5 μM. However, more than 90% of cells survived the concentration of 10 μM in MCF-7/DOX cell treated with DOX for 24 h (Figure 1D). Based on this resistance testing result, we then examined the cytotoxicity of C49 in MCF-7/DOX cell by CCK-8 assay at 48, and 72 h. C49 under concentration of 12.5 μmol/L was nontoxic through calculation based on IC_20_ value. Thus, this dose was applied to the subsequent experiment to eliminate C49 toxicity interfering with drug-resistant cell strains.

### Effect of C49 Combined With DOX on Proliferation of MCF-7/DOX Cells

**FIGURE 2:**
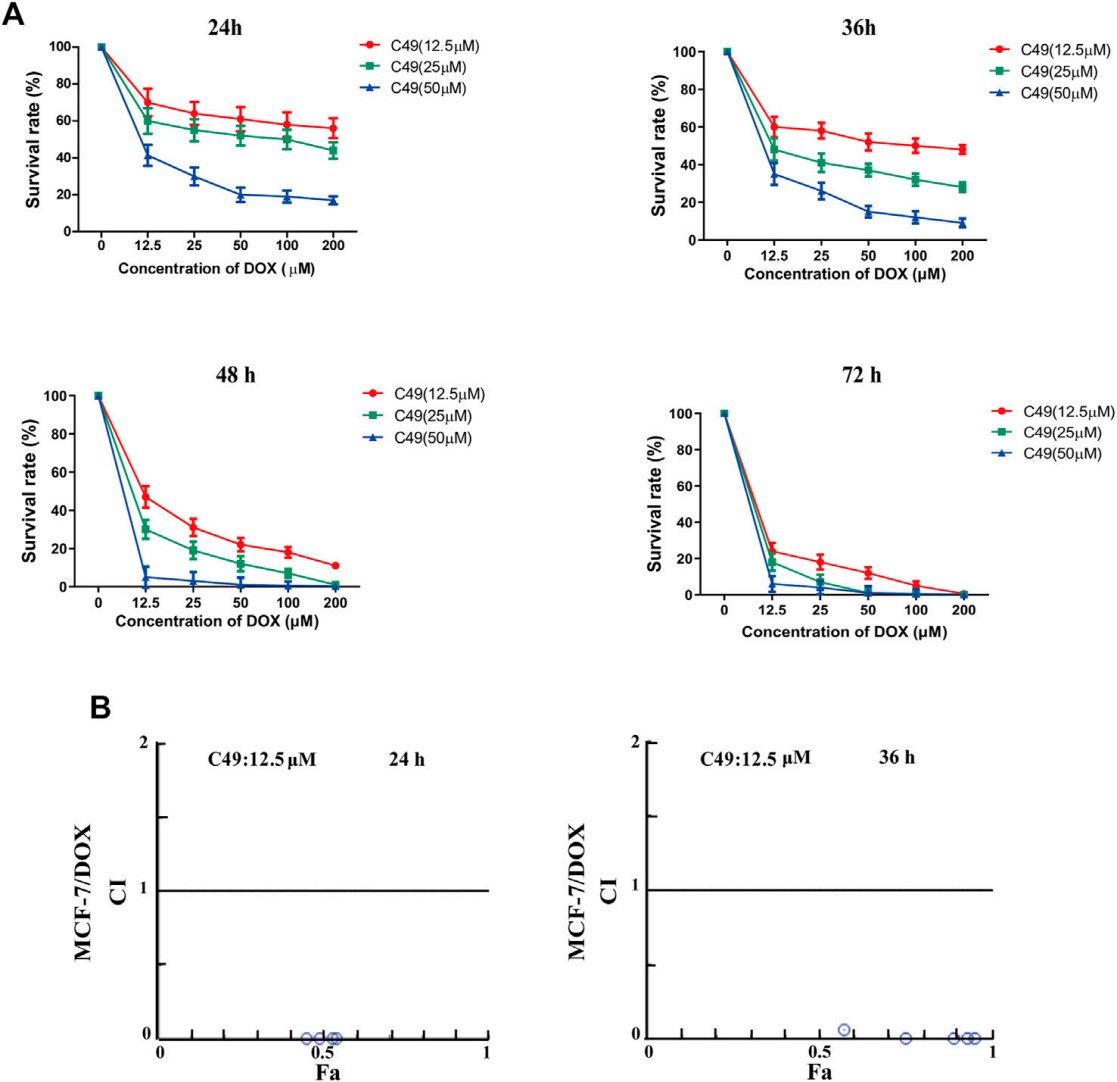
The effect of C49 combined with DOX on the viability of MCF-7/DOX cell. **(A)** DOX or C49 combined with DOX inhibits the viability of MCF-7/DOX cells. Each point represents mean ± SD, *n* = 3. *p < 0.05, **p < 0.01 and ***p < 0.001 for DOX treated cells vs. combination treated cells. **(B)** Compusyn software used to compute the Combination Index (CI). CI values <1, =1, and >1 respectively represents synergic, addictive and antagonistic effect.

MTT assay was performed to assess the proliferation of cells treated with different concentration of C49 and DOX. After combined treatment of C49 and DOX on MCF-7/DOX cells for 24, 36, 48 and 72 h, the anti-proliferative effect was remarkably lower in DOX single drug treatment group than that of C49 combined with DOX group. Compared with C49 (12.5 μM) combined with DOX group, the anti-proliferative effects of 25 and 50 μM C49 combined with DOX groups were markedly stronger than that of C49 low-dose group. The effects were dose and time dependent (Figure 2A). This result indicated that C49 could reverse the resistance of MCF-7/DOX cells against DOX, and a high C49 concentration is associated with a strong effect of reversing drug resistance. Meanwhile, the synergistic index of 12.5 μM C49 and DOX was evaluated using Calcusyn software. The CI values were smaller than 1 after their combined action for 24 and 36 h, suggesting that C49 and DOX exerted synergistic effect (Figure 2B).

### C49 Enhanced the Cytotoxicity of Doxorubicin to Repress Cell Proliferation and Induced Cell Apoptosis

**FIGURE 3:**
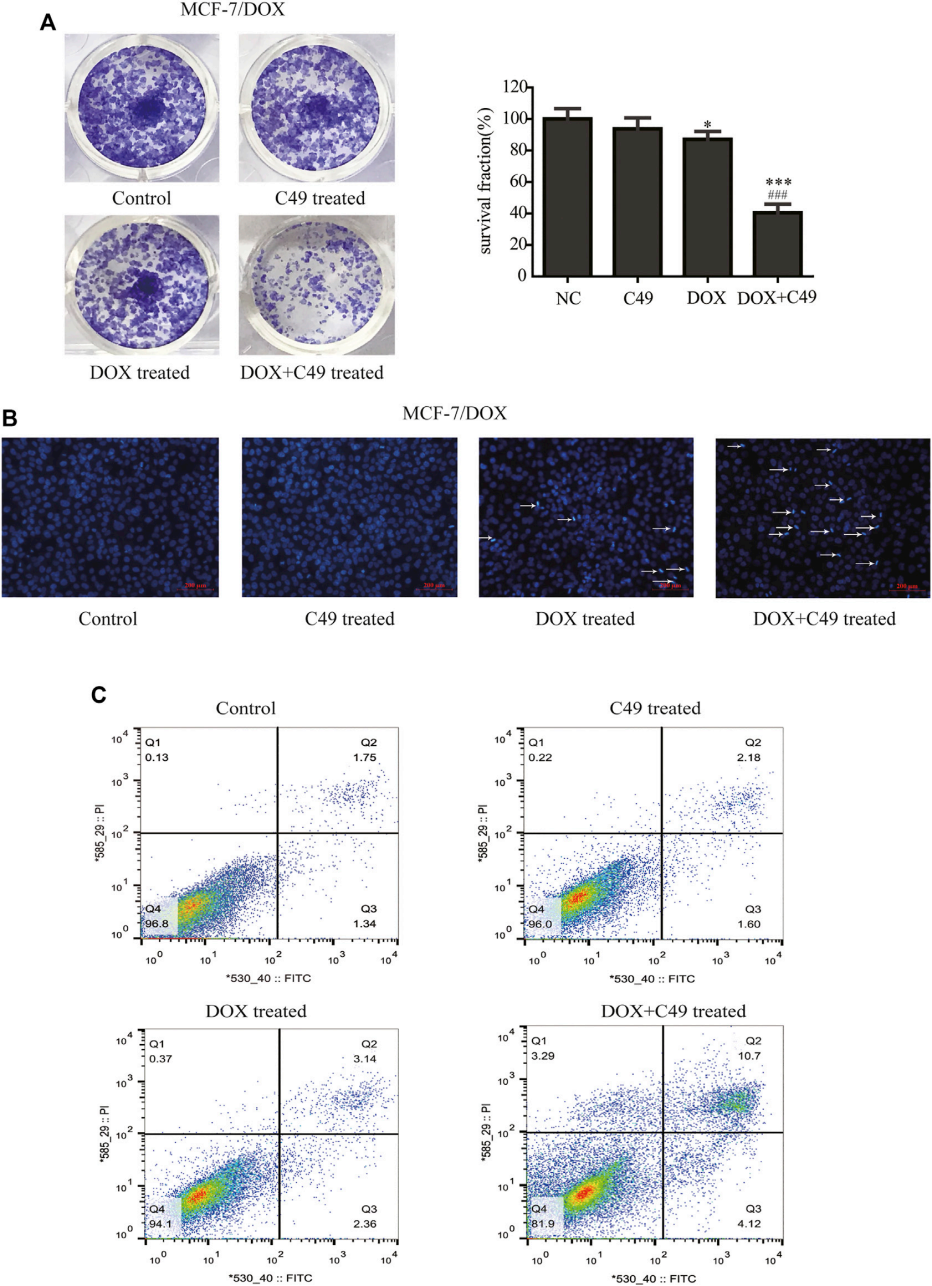
C49 combined with DOX inhibited proliferation and promoted apoptosis of MCF-7/DOX cells. **(A)** Colony formation showed the proliferation of MCF7/DOX cells after DOX (8 μM) and/or C49 (12.5 μmol/L) treatment. The right panel was the quantitative analysis of colony formation experiment. Each bar represents mean ± SD, *n* = 3. *p < 0.05, **p < 0.01 and ***p < 0.001 for control cells vs. DOX and/or C49 treated cells. ^###^ *p* < 0.001 for DOX treated cells vs. combination treated cells. **(B)** Hoechst 33,258 staining showing nuclear morphology of MCF-7/DOX cells 24 h after DOX (8 μmol/L) and/or C49 (12.5 μM) treatment. **(C)** Apoptotic cells detected by flow cytometry after DOX (8 μM) and/or C49 (12.5 μM) treatment for 48 h.

Colony formation test, Hoechst 33,258 staining method, and FCM were used to examine the proliferation inhibitory effect of DOX, C49 (12.5 μM) or combination of both on MCF-7/DOX cells. Figure 3A showed that colony formation has no significant difference in C49 treatment group (*p* > 0.05). However, there was a difference in colony formation in DOX treatment group (*p <* 0.05) and significant difference was observed in C49 combined with DOX treatment group (*p <* 0.01) compared with the blank control group. Therefore, DOX and C49 in combination with DOX treatment could inhibit the proliferation of MCF-7/DOX cells, and the inhibiting effect of C49 combined with DOX group was remarkably stronger than that of DOX alone.

The cell nuclear morphology of the four groups were observed using Hoechst 33,258 staining at 48 h after cells treated with drugs. Usually, cells exhibited apoptotic features with chromatin condensation, nuclear condensation and DNA fragmentation (Wang et al., 2011; Li et al., 2018). As shown in Figure 3B, the nuclear morphology of cells treated with C49 did not undergo marked changes, the cells treated with DOX showed increasing chromatin condensation and nuclear fragmentation, and a large number of cells treated with C49 combined with DOX are dying compared with cells in the blank control group. This result indicated that C49 combined with DOX could strengthen the DOX cytotoxicity in MCF-7/DOX cells. The cell apoptosis were detected by FCM. As shown in Figure 3C, the percentage of apoptotic cells (including the early and late apoptotic cells) was 1.05% in the control group, 3.23% in C49 group, 8.48% in DOX group and 57.4% in C49 combined with DOX group, indicating that the C49 and DOX alone had weak effect causing apoptosis of MCF-7/DOX cells, but C49 could enhance the sensitivity of DOX in MCF-7/DOX cells. These results suggested that C49 did not have marked cytotoxicity but could reverse drug resistance and enhance the cytotoxicity of DOX inhibit cell proliferation and induce cell apoptosis.

### C49 Increased Intracellular Accumulation of Doxorubicin in MCF-7/Doxorubicin Cells

**FIGURE 4:**
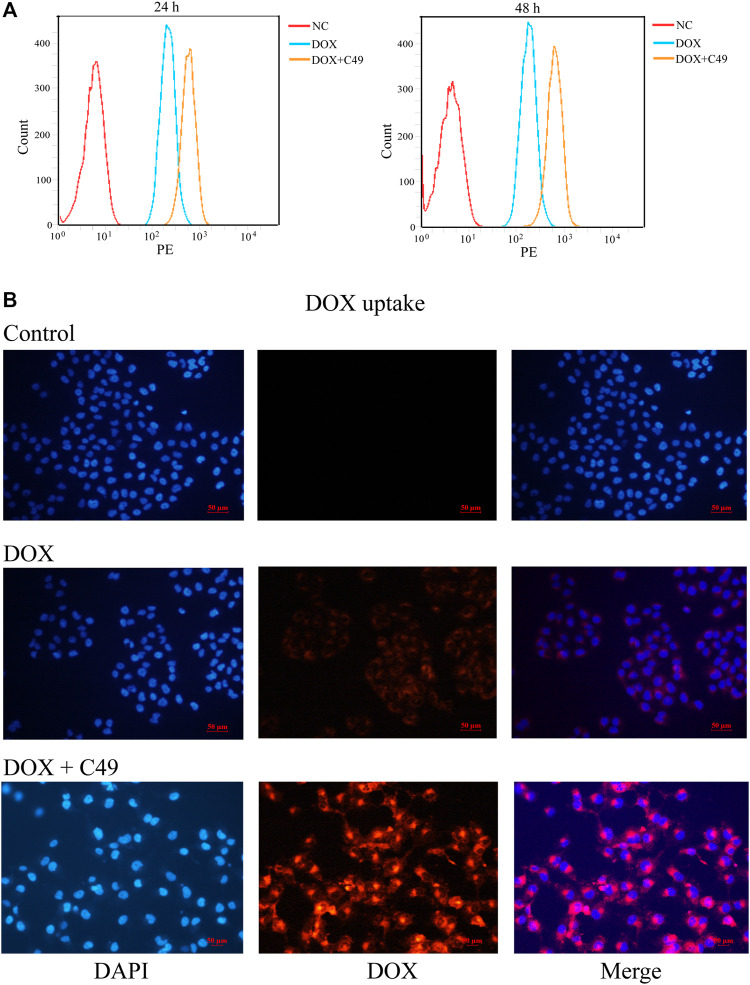
C49 combined with DOX increased intracellular concentration of DOX in MCF-7/DOX cells. **(A)** Cells were treated with DOX (8 μM) and C49 (12.5 μM) for 24 or 48 h, and DOX accumulation in the cells were analyzed using flow cytometry. **(B)** Cells were treated with DOX (8 μM) and/or C49 (12.5 μM) for 48 h and stained with DAPI. DAPI: blue fluorescence, DOX: red fluorescence.

FCM assay and immunofluorescence (IF) assay were performed to examine the intracellular accumulation of DOX in MCF-7/DOX cells. Cells were treated with DOX or C49 combined with DOX for 24 and 48 h. FCM was adopted to detect intracellular DOX concentration. As shown in Figure 4A, intracellular DOX concentration in cells of DOX group was increased compared with that in the control group, and DOX concentration in C49 combined with DOX group was the highest among the three groups. This result indicated that C49 could facilitate intracellular accumulation of DOX in MCF-7/DOX cells. Immunofluorescent microscope was used to verify the result. As shown in Figure 4B, red fluorescence was increased in MCF-7/DOX cells in DOX (8 μM) group relative to the control group, while that in C49 (12.5 μM) combined with DOX (8 μM) group was significantly increased relative to DOX group. These results suggested that C49 increased the intracellular concentration of DOX in MCF-7/DOX cells.

### Differential Gene Expression Between MCF-7 and MCF-7/DOX Cells

**FIGURE 5:**
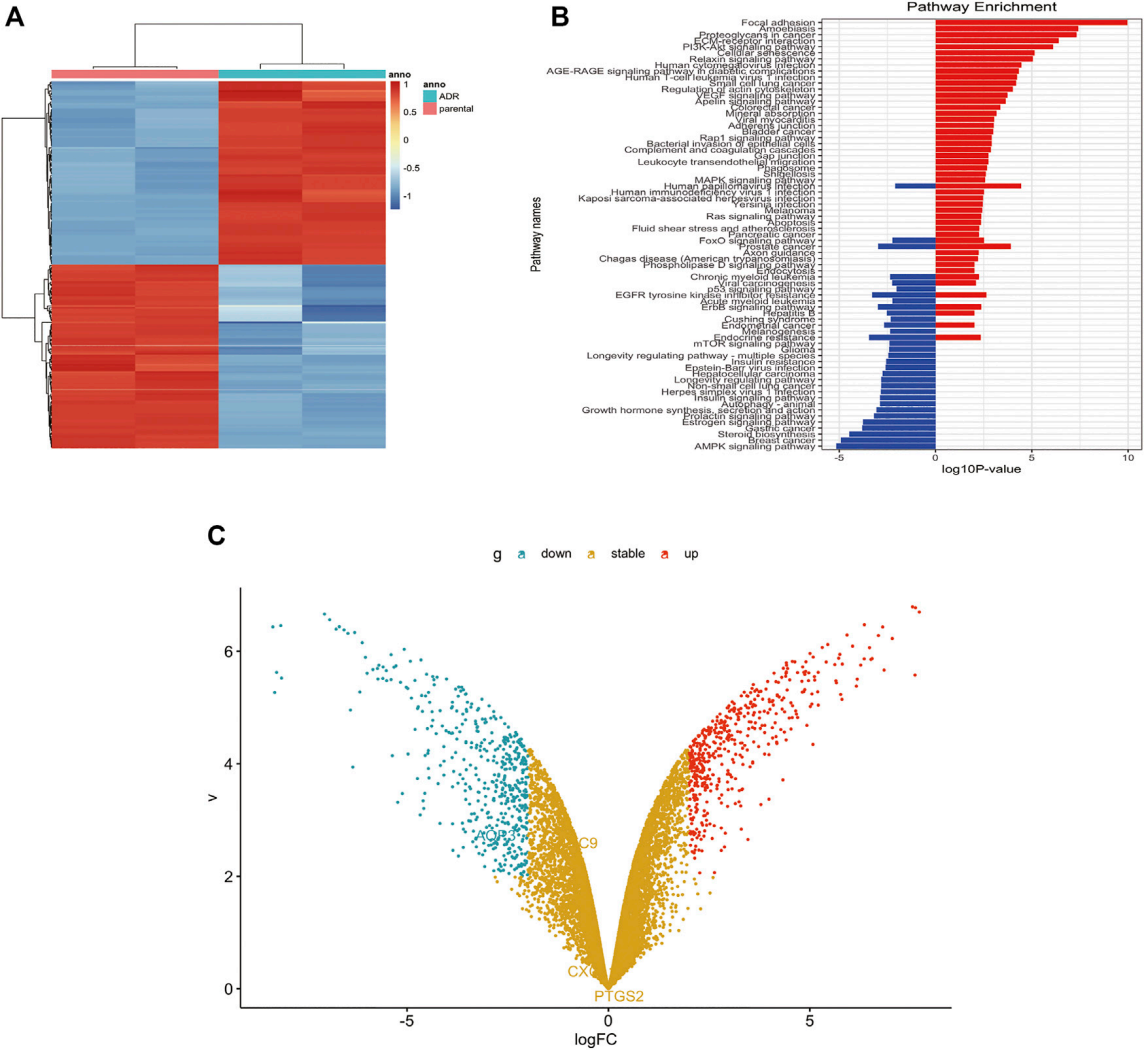
P-gp upregulated in MCF-7/DOX cells. **(A)** Heatmap of differentially expressed genes in GEO dataset. **(B)** Analysis of pathways related to drug resistance revealed the PI3K/Akt signaling pathway is the fifth largest factor. **(C)** Volcano plot analyzed the differentially expressed gene between MCF-7 cells and MCF-7/DOX cells. The fold change threshold was >2, *p* value was <0.05.

GSE24460 gene chip was analyzed using Limma software package, Hochberg False Discovery Rate and Benjamini. Differential genes were screened through the expression of various chips, such as ABCB1, VIM, LDHB, NNMT, MMP1, FSTL1, CCN2, GPX1, ESR1, and AGR2 (Supplementary Table S1). Through the expression levels of the first 200 differential genes, heat maps were drawn to indicate differences of genes between the two cell lines (Figure 5A). KEGG graph showed that PI3K/Akt signaling pathway was activated in MCF-7/DOX cells (Figure 5B). Volcano plots embodied gene differences between the two cell lines (Figure 5C). The above-mentioned data indicated that PI3K/Akt signaling pathway and ABCB1 played dominant roles in the drug resistance of MCF-7/DOX cells.

### C49 Combined With Doxorubicin Repressed the Expression of P-gp, Phosphorylated-Phosphoinositide-3-kinase, and Phosphorylated-Protein Kinase B (p-Akt) proteins

**FIGURE 6:**
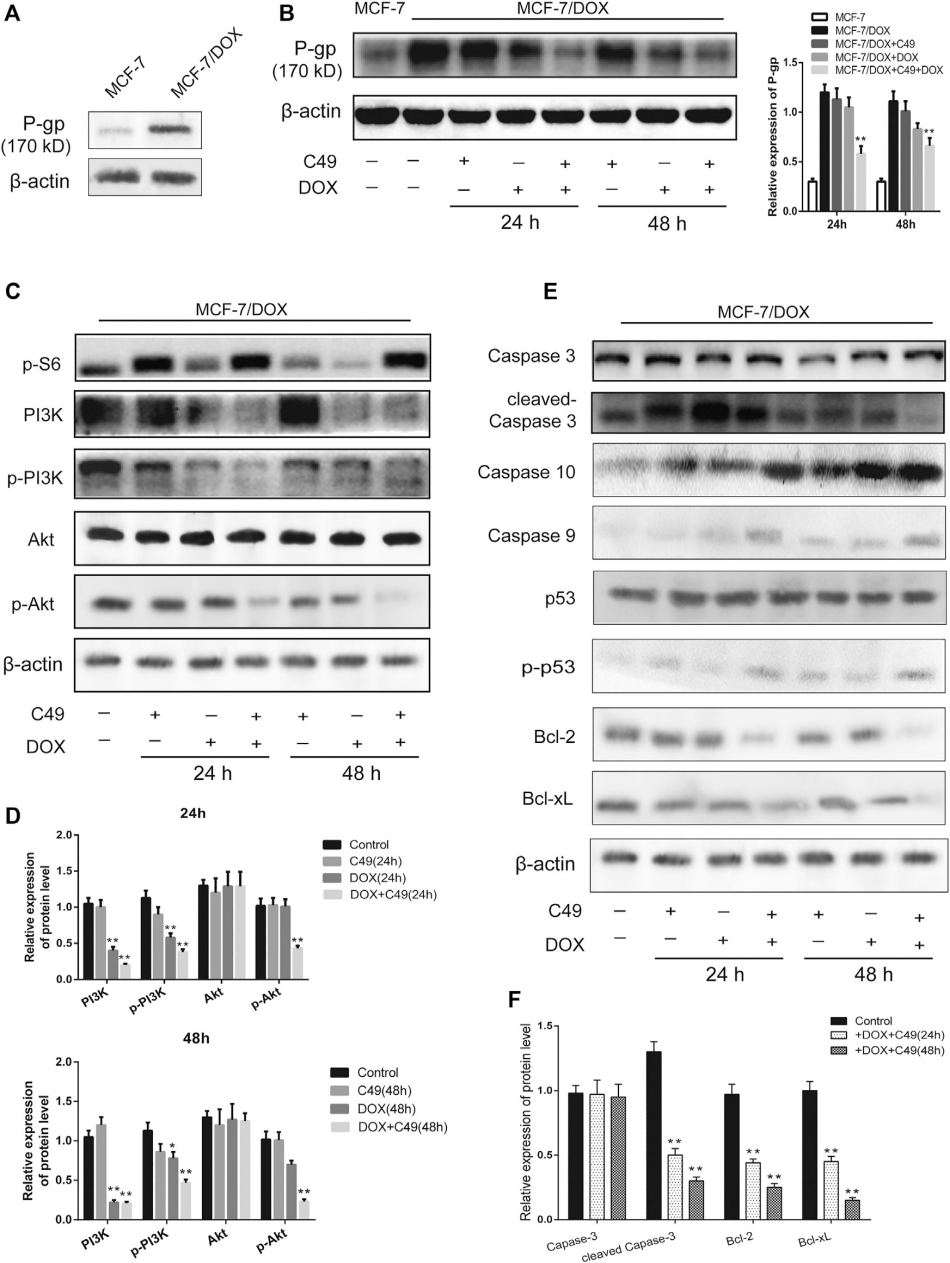
The combination of C49 and DOX inhibited P-gp expression, apoptotic signaling pathway and the PI3K/Akt signaling pathway. **(A)** The P-gp expression in MCF-7 cells and MCF-7/DOX cells. **(B–F)** WB and quantitative analysis of P-gp, PI3K, p-PI3K, Akt, p-Akt, Caspase-3, Caspase-10, Caspase-9, p53, p-p53, Bcl-2, and Bcl-xL proteins expression compared with β-actin. *p < 0.05, **p < 0.01*vs.* MCF-7/DOX cells.

Western blot analysis was used to detect the changes of protein expression in MCF-7/DOX cells before and after drug intervention. The results showed that the expression of P-gp protein in MCF-7/DOX cells was markedly higher than that in MCF-7 cells (Figure 6A). Following drug intervention for 24 and 48 h, C49 (12.5 μM) combined with DOX (8 μM) could remarkably reduce the level of P-gp protein in MCF-7/DOX cells compared with the control group (Figure 6B). As shown in Figure 6C, the expression levels of p-PI3K and p-Akt proteins in the combinational treatment group were significantly reduced, but the expression levels of total-PI3K and total-Akt did not obviously changed. The expression of apoptins in the downstream of PI3K/Akt signaling pathway was explored, and the results showed that the expression of Caspase-3 and Caspase-9 in the combinational treatment group were remarkably up-regulated while the expression of Bcl-2 was markedly down-regulated (Figure 6D). Some reports have shown an increase in phosphorylated p53 was induced by treatment with chemotherapeutic reagents *in vitro* and in murine xenograft models (Ohara et al., 2018). In our present study, there is no change in the expression level of p53 among the different groups, but its phosphorylated expression have been changed after C49 treatment in MCF-7/DOX cells (Figure 6D). The corresponding semi-quantitative results are showed in Figure 6E. Therefore, C49 combined with DOX could reverse MDR by repressing the expression of P-gp protein and PI3K/Akt signaling pathway.

### The Role of C49 on Breast Cancer Xenograft Mice

**FIGURE 7:**
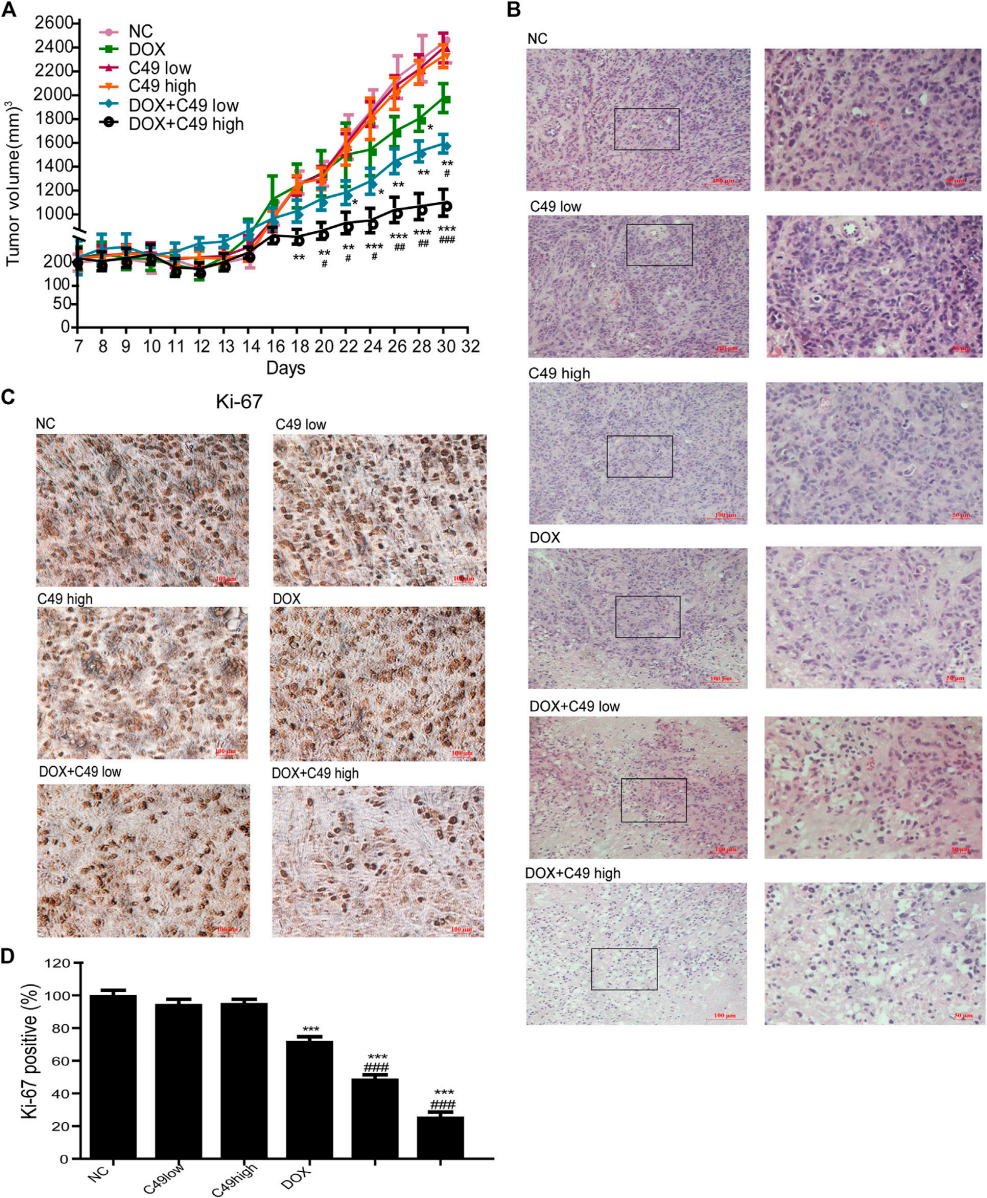
C49 combined with DOX increased antitumor activity in MCF-7/DOX bearing nude mice. **(A)** Tumor volume in each group measured once every two days during drug treatment. Each bar represents mean ± SEM. Significant differences between control and DOX and/or C49 drug treatment group were indicated by *p < 0.05, **p < 0.01 and ***p < 0.001. Significant differences between DOX treatment group and combination treatment group were indicated by ^#^ *p* < 0.05, ^##^ *p* < 0.01 and ^###^ *p* < 0.001. **(B)** H&E staining showed the necrosis of the tumor xenografts. **(C)** Ki-67 immunostaining showed the proliferation of tumor xenografts. **(D)** Quantitative analysis of Ki-67 immunostaining assay.

A breast cancer xenograft model was constructed on nude mouse to further verify the effect of C49 on the reversal of MDR *in vivo*. Consistent with previous results, no difference was noted in animal weight and hepatorenal toxicity among treatment groups during the experiment (Supplementary Figures S1, S2). After C49 or C49 combined with DOX intervention, C49 low and high-dose groups did not exert the effect of inhibiting tumor growth, DOX treatment group had a better inhibiting effect on tumor growth, and low and high-dose groups of combinational treatment remarkably inhibited tumor growth in a dose-dependent way compared with the control group (Figure 7A). H&E staining was implemented to detect the pathological change of tumor tissues in these mice. As shown in Figure 7B, tumor cells disorganization was evident in the control group with disorder cell arrangement and pathological nuclear mitotic figures. Compared to cells in the control group, those in the combination treatment group were sparsely arranged. Apart from nuclear fragmentation and dissolution, small vacuoles could be observed in the cytoplasm of many tumor cells. Nuclear fission was remarkably reduced. The cell proliferation of tumor tissues was examined by IHC staining. The expression of Ki-67 in tumor tissues was the highest in the control group, and lowest in the combinational treatment group compared to the control group (Figure 7C). These results showed that C49 had no significant antitumor effect but could significantly reverse the DOX resistance in grafted tumor of drug-resistant breast cancer. C49 combined with DOX could remarkably enhance the cytotoxicity of DOX to repress tumor growth and could thus realize the antitumor effect.

## Discussion

The main treatment for breast cancer is surgery, which can lengthen the overall survival of patients when combined with chemotherapy. DOX is a first-line chemotherapeutic drug for breast cancer, which can kill tumor cells but it causes MDR if used for a long period of time, resulting in chemotherapy failure. Konieczkowski et al. (Konieczkowski et al., 2018) found that the mechanism of DOX in inducing MDR involved multiple processes, e.g., reducing drug absorption, increasing drug excretion and changing drug metabolism, where high expression of P-gp protein was the primary cause of drug resistance of tumors (Gao et al., 2018; Lowrence et al., 2019). In DOX-resistant breast cancer cells, P-gp protein can pump DOX out of MCF-7/DOX cells, as a result, DOX fails to exert effective cytotoxic effect (Cao et al., 2019) and breast cancer cells can evade attack of chemotherapeutics (Genovese et al., 2017). Reversal of drug resistance can be realized by repressing P-gp protein. For example, cryptotanshinone and dihydrotanshinone of *Salvia miltiorrhiza* can repress expression of P-gp protein to reverse drug resistance (Lee et al., 2018). Myrsinol diterpene can inhibit the excretory function of P-gp protein to reverse drug resistance of breast cancer (Wang et al., 2016). In our study, C49 combined with DOX could lower the expression of P-gp protein, leading to intracellular DOX accumulation in drug-resistant cells, inhibit cell proliferation and promote cell apoptosis.

MDR is associated with abnormal activation of relevant signaling pathways in drug-resistant cells. PI3K/Akt plays a critical role in regulating cell proliferation, survival, metabolism, and apoptosis of normal cells (Aoki et al., 2017; Tewari et al., 2019). This signaling pathway is abnormally activated in various tumors, e.g., breast, lung, ovarian, and prostate cancers (Lu et al., 2016; Gu et al., 2018; Ediriweera et al., 2019; Verret et al., 2019; Madsen et al., 2020) and participates in mediating tumor MDR (Guerrero-Zotano et al., 2016; Yang et al., 2017; Li et al., 2019a; Liu et al., 2020). For example, PI3K/Akt signaling pathway is involved in paclitaxel resistance when it is abnormally activated in prostate cancer. Meanwhile, abnormally activated PI3K/Akt signaling pathway can contribute to the phosphorylated activation of serine sites of Bcl-2 protein and change its spatial conformation to discourage apoptins from exerting normal functions and inhibiting cell apoptosis.

Previous studies show that the expression of some protein molecules in PI3K/Akt signaling pathway is up-regulated in chemotherapeutic-resistant cells, and the drug-resistant phenotypes could be reversed by inhibiting those proteins. For example, resveratrol and matrine can reverse the MDR of breast cancer by repressing PI3K/Akt signaling pathway (Chen et al., 2018; Zhou et al., 2018). Likewise, PI3K/Akt can target the anti-apoptotic genes like Bcl-2 and Bcl-xL, resulting in the suppression of apoptosis machinery. A report confirmed that a natural chalcone, has been shown to exert growth inhibitory effects on the human Hepatocellular carcinoma cells serves as a transcription suppressor of anti-apoptotic genes like Bcl-2 and Bcl-xL (Ji et al., 2019). In yet another study, Xanthohumol caused arrest of the cancer cells at the G2/M phase of the cell cycle, which was also accompanied with suppression of Caspase-family (Liu et al., 2019). Our study showed that the expression of p-PI3K, p-Akt and Bcl-2 proteins reduced remarkably in MCF-7/DOX cells treated with C49 in combination with DOX, indicating that PI3K/Akt signaling pathway was involved. In contrast, those of p-p53, Caspase-9, Caspase-3, and Caspase-10 were elevated. Therefore, this signaling pathway can promote or inhibit downstream signaling molecules, such as Bcl-2, Caspase-9 and p-p53, to regulate cell apoptosis.

In the DOX-resistant breast cancer xenograft mouse model, different concentrations of C49 or C49 combined with DOX were used for the treatment of tumors. The results showed that C49 combined with DOX could remarkably repress tumor growth, inhibit tumor cell proliferation, and accelerate tumor cell apoptosis. suggesting that C49 can enhance the chemotherapeutic effect of DOX *in vivo*.

## Conclusion

C49 combined with DOX can remarkably inhibit the proliferation of MCF-7/DOX cells and promote cell apoptosis both *in vitro* and *in vivo*. C49 may degrade the ability of P-gp protein to pump DOX out of cells by repressing the expression of P-gp protein, increasing the intracellular concentration of DOX. The combination of C49 and DOX may also repress the expression of p-PI3K and p-Akt proteins and reverse the drug resistance of breast cancer. Therefore, C49 can be a potential therapeutic drug for reversing DOX resistance in the treatment of breast cancer and other cancers.

## Data Availability

The original contributions presented in the study are included in the article/Supplementary Material, further inquiries can be directed to the corresponding authors.

## Glossary

Akt: protein kinase B
Bcl-2: B cell lymphoma/leukemia-2
Bcl-xL: B cell lymphoma-extra large
BSA: bovine serum albumin
Caspase-10: cysteinyl aspartate-specific proteinase -10
Caspase-3: cysteinyl aspartate-specific proteinase -3
Caspase-9: cysteinyl aspartate-specific proteinase -9
CI: combination index
DAPI: 2-(4-Amidinophenyl)-6-indolecarbamidine dihydrochloride
DMSO: dimethyl sulfoxide
DOX: doxorubicin
FACS: fluorescence activating cell sorter
FBS: fetal bovine serum
FCM: flow cytometry
FITC: fluoresceine isothiocyanate
GEO: gene expression omnibus
H&E: hematoxylin and eosin
IC: inhibitory concentration
IC_20_: twenty percent of maximal inhibitory concentration
IC_50_: half maximal inhibitory concentration
IF: immunofluorescence
IHC: immunohistochemistry
KEGG: kyoto encyclopedia of genes and genomes
MCF-7: michigan cancer foundation-7
MDR: multidrug resistance
MTT: 3-(4,5-Dimethylthiazol-2-yl)-2,5-diphenyltetrazolium bromide
PBS: phosphate buffer saline
P-gp: p-glycoprotein
PI3K: phosphatidylinositol 3-kinase
PVDF: polyvinylidene fluoride
RIPA: radio immunoprecipitation assay
RPMI1640: roswell park memorial institute 1640
SDS-PAGE: sodium dodecyl sulfate-polyacrylamide gel electrophoresis
TBST: tris-buffered saline and tween 20
WB: western blot
