# Supplementary material for: A New Chalcone Derivative C49 Reverses Doxorubicin Resistance in MCF-7/DOX Cells by Inhibiting P-Glycoprotein Expression
Source: Front Pharmacol. 2021 Apr 13;12:653306. doi: 10.3389/fphar.2021.653306 (PMC8076869; doi:10.3389/fphar.2021.653306)
Supplement: Supplementary file 1 [file datasheet1.docx]

**Supplementary Figure**


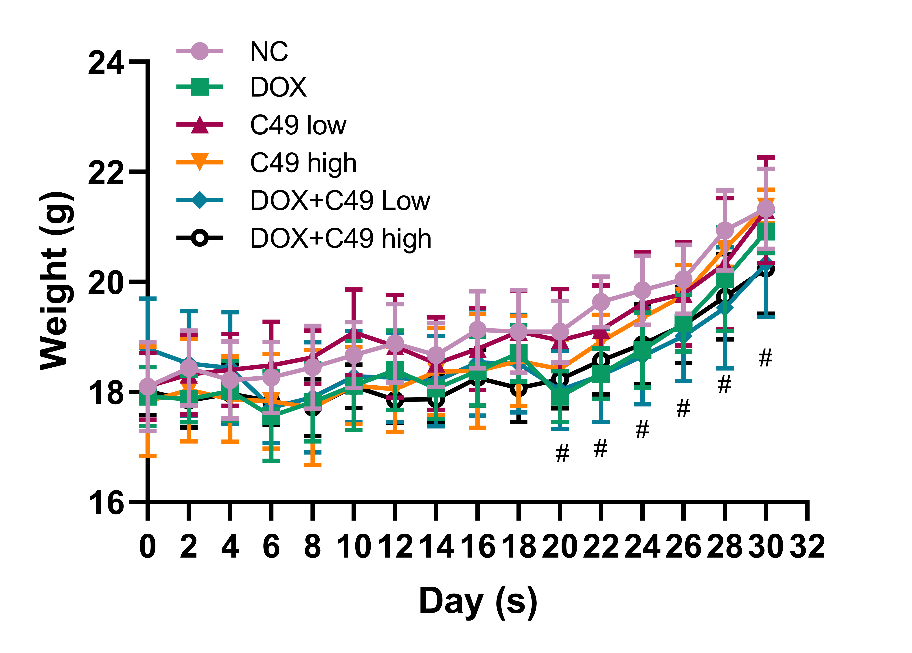


**Supplementary Figure 1 The effect of C49 on body weights of breast cancer xenograft mice**

Body weights of nude mice that were orally treatment with C49 for 24 days.

| Control | **C49** |
| --- | --- |
| 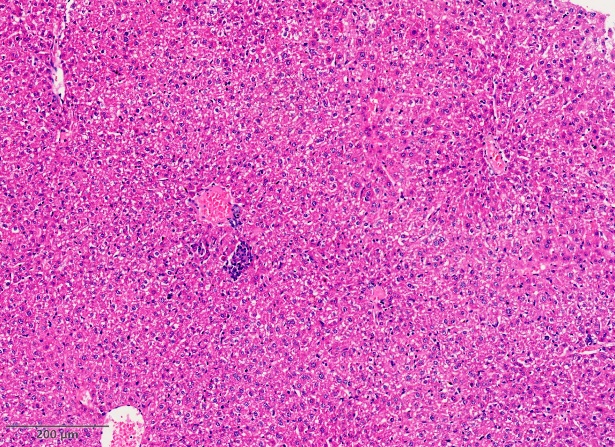 | 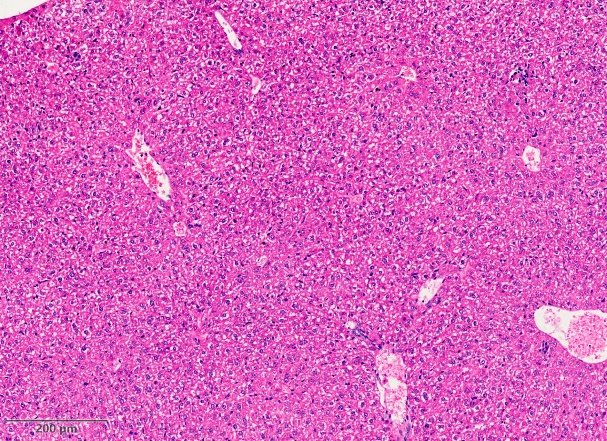 |

**Supplementary Figure 2 The effects of C49 on the liver in breast cancer xenograft mice**

The mice are from the model of breast cancer xenograft mice. Liver were stained with HE and observed under phase-contrast microscope. Scale bars, 200 μm.
